# Influence of Acupuncture Stimulation on Cerebral Network in Functional Diarrhea

**DOI:** 10.1155/2013/975769

**Published:** 2013-12-26

**Authors:** Siyuan Zhou, Fang Zeng, Jixin Liu, Hui Zheng, Wenjing Huang, Ting Liu, Dashuai Chen, Wei Qin, Qiyong Gong, Jie Tian, Ying Li

**Affiliations:** ^1^Acupuncture and Tuina School, The 3rd Teaching Hospital, Chengdu University of Traditional Chinese Medicine, No. 37 Shi'er Qiao Road, Chengdu, Sichuan 610075, China; ^2^School of Life Science and Technology, Xidian University, Shaanxi 710071, China; ^3^The institute for Social Medicine, Epidemiology and Health Economics, Charité University Medical Center, 10117 Berlin, Germany; ^4^Department of Radiology, The Center for Medical Imaging, Huaxi MR Research Center, West China Hospital of Sichuan University, Chengdu, Sichuan 610041, China

## Abstract

Acupuncture is a commonly used therapy for treating functional diarrhea (FD), although there is limited knowledge on the mechanism. The objectives of this study were to investigate the differences in brain activities elicited by acupuncture between FD patients and healthy controls (HC) so as to explore the possible mechanism. Eighteen FD patients and eighteen HC received 10 sessions of acupuncture treatment at ST25 acupoints. Functional magnetic resonance imaging (fMRI) scans were, respectively, performed before and after acupuncture. The defecation frequency, Bristol stool form scale (SBFS), and MOS 36-item Short Healthy Survey (SF-36) were employed to evaluate the clinical efficacy. After acupuncture, the FD patients showed a significant decrease in defecation frequency and BSFS score. The regional homogeneity (ReHo) map showed a decrease in the paracentral lobule and postcentral gyrus, and an increase in the angular gyrus, insula, anterior cingulate cortex (ACC), and precuneus in the FD group. Moreover, the changes in ReHo values in the ACC were correlated with the reduction in defecation frequency. Decreasing functional connectivity among the ACC, insula, thalamus, and orbital frontal cortex only existed in the FD group. Conclusively, acupuncture alleviated defecation frequency and improved stool formation in FD patients. The efficacy might result from the regulation of the homeostasis afferent processing network.

## 1. Introduction

Functional diarrhea (FD), one of the functional gastrointestinal disorders (FGID), is characterized by chronic diarrhea in the absence of structural or biochemical abnormalities that explain the symptoms [1]. According to the 2006 Rome III criteria, FD is defined as loose or watery stools without pain occurring in at least 75% of the stools [2]. The prevalence of FD ranges from 1.72% to 3.7% [3–5]. FD significantly influences the quality life of patients and consumes many healthcare resources [6]. Because of the unclear etiology and pathogenesis, the therapeutic options for FD are limited. As a result, complementary or alternative therapies are attractive to both patients and practitioners.

Acupuncture, an important traditional Chinese medicine (TCM) therapy, has been used to treat gastrointestinal symptoms for centuries in China. Now it has been increasingly accepted as a complementary and alternative treatment for functional gastrointestinal disorders in western countries [7, 8]. ST25 (Tianshu), an important acupoint in the stomach meridian of the Foot Yangming, is commonly used to treat intestinal illnesses, such as constipation, diarrhea, abdominal pain, and bloating [9]. In recent years, a large amount of clinical and animal studies proved that puncturing at ST25 is able to modulate gastrointestinal motility and increase the threshold of visceral sense and regulate gastrointestinal hormones [10–12].

Altered perceptual responses and reflex within the brain-gut axis have been proposed as a generally accepted model to explain the cardinal symptoms of FGID [13]. The gut and the brain are highly integrated and communicated bidirectionally. Within the brain, the gut is controlled mainly by the limbic system, a region responsible for both the internal and external homeostasis of the body [14]. On the other hand, previous research has shown that acupuncture stimulation mediated the limbic-paralimbic-neocortical network and somatosensory brain regions [15]. Neuroimaging technologies provide means of objective and visualization for exploring central mechanisms, which is significant in the acupuncture. Therefore, it is helpful to observe the mechanism of acupuncture by neuroimaging technologies.

We hypothesize that if acupuncture therapy is effective, it would improve gut function and couple with regulation of disease-related brain regions. In this study, we sought to investigate the differences in brain activity between FD patients and healthy controls (HC) after acupuncture using functional mapping with fMRI. We first identified regions showing different brain regional homogeneity (ReHo) after acupuncture in FD patients compared with controls. Then, we tested whether some of these regions were correlated with symptom changes. Finally, we explored functional connectivity alterations after acupuncture in both FD and HC.

## 2. Methods

### 2.1. Participants

Eighteen FD patients were recruited from September 2011 to December 2012. The inclusion criteria were as follows. (1) Patients were right-handed and aged between 20 and 30 years; (2) they matched the Rome III diagnosis criteria for FD [2]; (3) they did not use gastrointestinal drugs for one week before enrollment in the study; (4) they were not participating in other clinical trials; and (5) they signed an informed-consent form. The exclusion criteria were as follows. (1) Patients had diarrhea caused by organic disorders; (2) they suffered psychosis or bleeding disorders; (3) they suffered serious disease of the heart, liver, kidneys, or other severe illnesses; (4) suffered from severe depressive or anxiety symptoms (the scores of the Self-rating Depression Scale (SDS) or Self-rating Anxiety Scale (SAS) were greater than 75); (5) they had a combination of severe headache or migraine relieved by bed rest or medicine or had a history of head trauma with loss of consciousness, (6) they had a combination of severe dysmenorrhea relieved by bed rest or medicine; (7) they were pregnant, preparing to be pregnant or lactating; or (8) had any contraindications to acupuncture or fMRI.

Eighteen age and sex-matched right-handed HC were recruited by advertisement. Each healthy volunteer was free from any gastrointestinal symptoms or sign and accepted a review of medical history and a physical examination to exclude disease carriers and medication users. All healthy volunteers signed the informed consent.

### 2.2. Acupuncture Intervention

**Figure 1:**
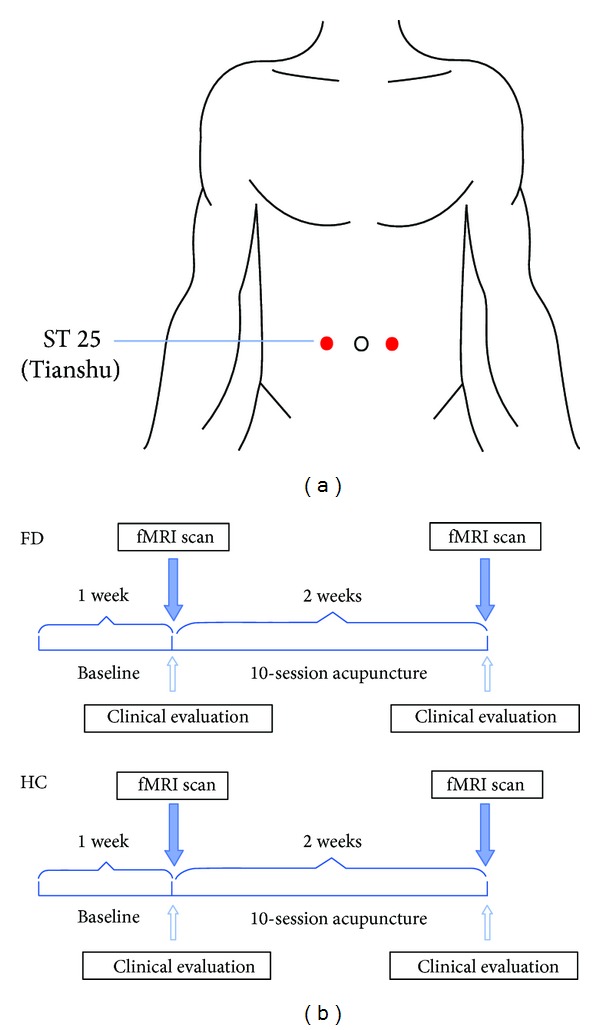
(a) Location of acupoints, (b) experimental paradigm.

Both FD patients and HC accepted 10 sessions total of acupuncture treatment over a period of 2 weeks (5 sessions per week). The acupuncture treatment was performed on ST25 (Tianshu), which are classical acupuncture points for diarrhea. The acupuncture points were punctured bilaterally (Figure 1). After the skin was prepped with alcohol, sterile disposable acupuncture needles (40–50 mm in length and 0.3 mm in diameter, Hwato, Suzhou, China) were inserted 30–40 mm deep and gently lifted, thrust, twisted, and rotated to achieve Deqi sensations (soreness, numbness, distention, and heaviness). The needles were retained for 30 minutes and manually manipulated every 10 minutes for 10–15 seconds to maintain the sensations. The treatment was performed by a licensed acupuncturist.

### 2.3. Outcome Measurement

The outcome in this study included the following items: the times of defecation and the score of the Bristol Stool Form Scale (BSFS) throughout the second week, the physical component summary (PCS) score and the mental component summary (MCS) score of the MOS 36-item Short Health Survey (SF-36) [16]. The times of defecation and the score of BSFS were used to assess gut symptoms. The BSFS with seven items (nut-like, lumpy sausage, sausage with cracks, smooth snake, soft blobs, mushy, watery) could be employed to monitor change in intestinal function [17]. Stool types 1 and 2 relate to constipation, while 6 and 7 relate to diarrhea. The SF-36, a commonly used questionnaire, is used for evaluating the quality of life. A higher PCS and MCS score mean better quality of life.

### 2.4. Magnetic Resonance Imaging Scanning

The experiment was carried out on a 3-T Siemens magnetic resonance scanner (Allegra; Siemens Medical System, Germany) at the MR Research Center of the West China Hospital at Sichuan University, Chengdu, China. The heads of the subjects were secured carefully with a comfortable holder, and ear plugs were used to reduce scanner noise. Prior to the functional run, a high-resolution structural image for each subject was acquired by the volumetric three-dimensional spoiled gradient recalled sequence (TR = 1900 ms; TE = 2.26 ms; data matrix, 256 × 256 mm^2^). Two expert radiologists examined the structural images of the participants to exclude the possibility of clinically silent lesions. The resting-state functional images were obtained with echo planar imaging (30 continuous slices with a thickness of 5 mm each; TR = 2000 ms; TE = 30 ms; flip angle, 90°; field of view, 240 × 240 mm^2^; data matix, 64 × 64; total volumes, 180).

### 2.5. Clinical Data Analysis

The clinical data were analyzed by the SPSS 16.0 statistical software (SPSS Inc., Chicago, IL, USA). All the numerical variables in this paper are presented as mean ± standard deviation (SD). Two independent-sample *t*-tests and Mann-Whitney *U* test were used to examine differences between FD group and HC group at the baseline (95% CI, 2-sided); paired sample *t*-test and Wilcoxon signed rank test were used to examine differences in both groups between before and after the acupuncture (95% CI, 2-sided). A *P* value < 0.05 was considered statically significant.

### 2.6. ReHo Analysis

Regional homogeneity (ReHo), a data-driven method, analyzes the blood oxygen level-dependent signals of the brain, which could help reveal the high complexity of the human brain function [18]. The first ten volumes were discarded to exclude nonequilibrium effects of magnetization and to allow subjects to adapt to the scanning environment. Data preprocessing was carried out using Statistical Parametric Mapping 5 (SPM5) (http://www.fil.ion.ucl.ac.uk/spm). First, the images were corrected for the acquisition delay between slices, aligned to the first image of each session for motion correction, and spatially normalized to the standard MNI template in SPM5. No subjects had head motions exceeding 1 mm of movement or 1° rotation in any direction. Then, a bandpass filter (0.01 Hz < *f* < 0.08 Hz) was performed to remove physiological and high-frequency noise [19]. After that, individual ReHo maps were generated by assigning each voxel a value corresponding to Kendall's coefficient of concordance (KCC) of its time series with its nearest 26 neighboring voxels [18]. Next, a whole brain mask was used to remove nonbrain tissues and noise on the ReHo maps, and individual ReHo maps were standardized by their own mean KCC within the mask [20, 21]. Finally, processed images were smoothed with an anisotropic Gaussian kernel (full width at half-maximum (FWHM), 6 mm) [18].

ReHo changes in FD patients or HC were determined by the contrast between baseline and after treatment. Statistical parametric maps were constructed by computing a paired* t*-test, which was defined as postacupuncture minus preacupuncture (family-wise error (FWE) correction at *P* < 0.05). The defecation frequency is the key index for evaluating the disease severity of functional diarrhea, and a lot of studies have demonstrated that the ACC played a key role in the regulation of gastrointestinal function. Consequently, the altered ReHo value in ACC area of FD was extracted and correlated with the decrease in defecation frequency during the second week (second week of the treatment minus baseline) to identify the association between brain response and symptoms changes.

### 2.7. Functional Connectivity Analysis

Functional connectivity has been used to describe the relationship between the neuronal activation patterns of anatomically separated brain regions, which reflect the level of functional communication between regions [22, 23]. We employed the functional connectivity method to assess resting-state properties after the acupuncture treatment. The left ACC was selected as the “seeding” region for the functional connectivity analysis, because ReHo analysis showed that the change of ReHo after acupuncture treatment in the left ACC had a significant negative correlation with the changes in defecation frequency. The preprocessing steps were performed according to a previous publication in SPM5, including realignment, normalization, bandpass filtering (0.01–0.08 Hz), and smoothing (FWHM = 6 mm) [24]. Functional connectivity was computed using an approach based on a seed voxel correlation [25]. For each subject, the correlation analysis was conducted between the seed reference and the rest of the whole brain in a voxelwise manner by regressing out the effects of head motion parameters. The resulting correlation was transformed to approximate Gaussian distribution using Fisher's *Z* transformation [24] and then analyzed with a paired sample* t*-test to identify voxels showing a significant correlation with the seed time series in FD or HC after acupuncture stimulation (FWE correction, *P* < 0.05).

## 3. Results

### 3.1. The Baseline Characteristics

There was an insignificant difference in the age and gender between the two groups, which were comparable (Table 1). Compared with HC, the FD patients demonstrated significantly more frequent defecations, higher BSFS scores, and lower PCS and MCS scores from the SF-36 (*P* < 0.01) (Table 1).

### 3.2. Effects of Acupuncture in FD and HC

In the HC group, the change in defecation frequency, BSFS score, PCS score, and MCS score were not significant (*P* > 0.05) after acupuncture (Table 2). In the FD group, the defecation frequency decreased from 16.17 ± 5.28 to 9.78 ± 3.75 (*P* < 0.001); the BSFS score decreased from 5.58 ± 0.63 to 4.75 ± 0.52 (*P* = 0.001); and the PCS score and MCS scores were not significant (*P* > 0.05) (Table 2).

### 3.3. Regional Homogeneity Changes

**Figure 2:**
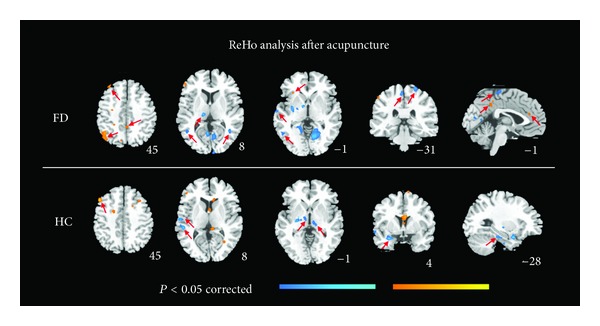
Regional homogeneity (ReHo) changes in functional diarrhea (FD) patients and healthy controls (HC) after acupuncture (*P* < 0.05, family-wise error corrected).

**Figure 3:**
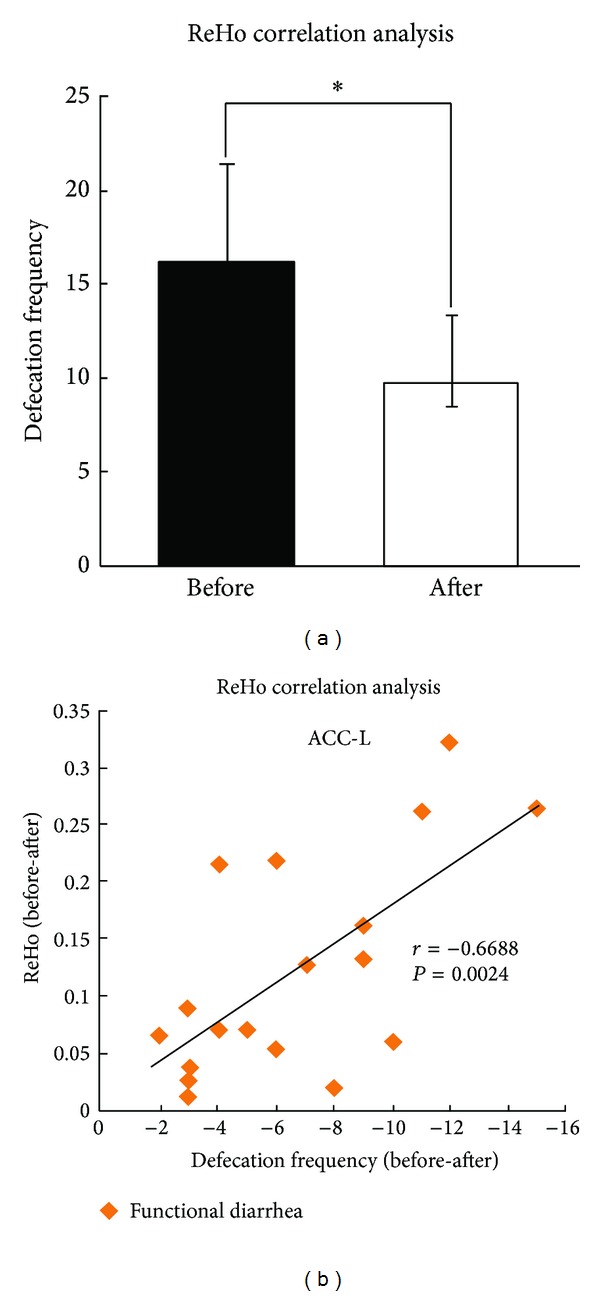
The correlation coefficients of Regional homogeneity (ReHo) and clinical variables. (a) In the functional diarrhea (FD) group, compared with preacupuncture, the defecation frequency postacupuncture was significantly decreased (*P* < 0.001). (b) In the FD group, the change in defecation frequency was significantly related to the ReHo increase in the left ACC. ACC: anterior cingulate cortex; L: left; *r*: correlation coefficient; *P*: *P value*; *P < 0.001.

In the FD group, a decrease in ReHo was observed in the middle temporal gyrus, paracentral lobule, postcentral gyrus, and thalamus, while an increase in ReHo was observed in the middle frontal gyrus, angular gyrus, insula, ACC, and precuneus (*P* < 0.05, FWE corrected) (Figure 2). In the HC group, ReHo decreased after acupuncture in the thalamus, amygdala, parahippocampus, middle temporal gyrus, and inferior temporal gyrus; it increased in the middle frontal gyrus (*P* < 0.05, FWE corrected) (Figure 2). Increased ReHo in the left ACC after acupuncture treatment was found to be negatively correlated (*r* = −0.6688, *P* = 0.0024) with the change in defecation frequency in the FD group (Figure 3).

### 3.4. Functional Connectivity Changes

**Figure 4:**
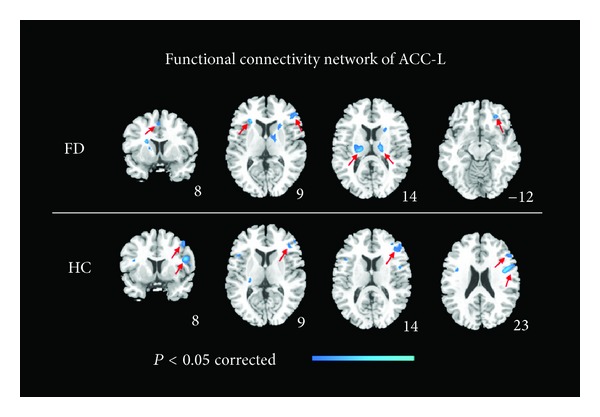
Functional connectivity network of ACC-L changes in functional diarrhea (FD) patients and healthy controls (HC) after treatment (*P* < 0.05, family-wise error corrected). ACC: anterior cingulate cortex; L: left.

In the FD group, the supplementary motor area, inferior frontal gyrus, orbital frontal cortex (OFC), thalamus, and insula were found to be negatively correlated with the left ACC after acupuncture (*P* < 0.05, FWE corrected) (Figure 4). Compared with preacupuncture, the precentral gyrus and inferior frontal gyrus of the HS group were negatively correlated with the left ACC (*P* < 0.05, FWE corrected) (Figure 4).

## 4. Discussion

In the present study, we investigated the cumulative effects of acupuncture stimulation on the resting-state brain activity of FD and compared them with HC. Clinical outcome showed that acupuncture was effective for the FD but had no effect on the HC. Neuroimaging results demonstrated the similarities and differences between FD and HC.

In this study, an increased ReHo in the middle frontal gyrus and a decreased ReHo in the thalamus and temporal cortex were found in both groups after acupuncture treatment. The middle frontal gyrus belongs to the dorsolateral prefrontal cortex (DLPFC), which is generally considered as a cognitive brain region. The DLPFC is directly interconnected with the sensorimotor cortex and indirectly connected with limbic structures that process internal information. The DLPFC is critical for arbitrary associations between sensory cues, rewards, and voluntary actions [26]. The major role of the thalamus is to modulate the flow of information to the cortex. It relays sensation, motor signals, and spatial sense to the cerebral cortex [27] and mediates the interaction of attention and arousal [28]. The middle temporal gyrus and inferior temporal gyrus are located on the lateral surface of the temporal lobe. Previous functional neuroimaging research has demonstrated that these areas are involved in cognitive processes such as semantic memory and language, as well as multimodal sensory integration [29, 30]. Both the FD group and HC group accepted the stimulation of inserting a needle underlying the muscle layer. The somatic afferent nerve in the skin and muscle were activated by acupuncture and produced somatic sensory information that was projected to the thalamus and brain cortex. Since all of these regions were altered in both groups associated with sensory information or cognition, we speculated that the alteration may be related to the response of the body to the acupuncture stimulation.

In this study, acupuncture stimulation elicited more extensive cerebral ReHo changes in the FD group as compared with the HC group. The increases in the angular gyrus, precuneus, ACC, and insula and the decrease in the paracentral lobule and postcentral gyrus were found only in the FD group but not in the HC group. Furthermore, the correlation analysis showed a negative relationship between the variation of ReHo in the ACC and the change in defecation frequency after acupuncture in the FD group. The ACC, insula, and somatosensory cortex were the main components of the visceral sensory neuromatrix, which is commonly described by the term “homeostatic afferent processing network” [13, 31]. The spinothalamic tract projects to the thalamus, from which information is relayed to the somatosensory cortices, the ACC, and the insula, respectively [32]. The somatosensory cortex provides information about intensity and localization of the visceral stimulus. The insula is the interoceptive cortex, where sensory information about the internal state of the body is processed. It plays a significant role in integrating emotional information and visceral sensation and involves a higher-order control of the autonomic visceromotor response [33, 34]. The ACC participates in encoding gastrointestinal sensory signals [35] and has several projection sites that may mediate visceromotor activity, such as the parasympathetic nucleus of the solitary tract, dorsal motor nucleus of the vagus, and sympathetic thoracic intermediolateral cell column [36]. Stimulation of the ACC could evoke visceromotor and autonomic activity changes in the animal [37, 38]. Sufficient evidence of neuroimaging has emerged to prove that functional gastrointestinal disorder patients are distinct from healthy people. The distinctions exist in the ACC, insula, or somatosensory cortex on a ReHo map [39], cerebral glycometabolism [40], grey matter density [41], cortical thinning [42, 43], and fractional anisotropy extracted from white matter regions [44]. Based on those properties, we considered that acupuncture could regulate brain regions which were associated with FD for rebalancing abnormal gut function. The angular gyrus and precuneus are part of the default mode network (DMN) that are active in the resting state but become deactivated when exposed to externally oriented attention. The DMN affects homeostasis and the ability to appropriately regulate internal experiences such as body state, feelings, and emotions [45]. In the normal people, the DMN could provide a balance of opposing forces to enhance the maintenance of information for interpreting, responding to, and even predicting environmental demands [46]. Recent studies have already demonstrated that the DMN is associated with Alzheimer's disease [47], schizophrenia [48], depression [49], attention deficit hyperactivity disorder [50], and chronic pain [51]. These suggested that the DMN is different in healthy controls and patients. In our study, the increase in ReHo in these regions, which only happened in the FD group, could relate to the unbalanced physical state. DMN might be more active in patients after acupuncture to maintain homeostasis.

As ReHo of the left ACC increased after acupuncture and showed a negative correlation with the change in defecation frequency, we chose this region as the region of interest (ROI) to investigate the resting-state functional connectivity change after acupuncture stimulation. Both groups showed significantly decreased functional connectivity in the left ACC to the PFC and motor cortex compared with preacupuncture. Besides, decreased functional connectivity in the left ACC was also found in insula, thalamus and OFC in the FD group. The extent of structural links between the ACC and the lateral PFC and motor cortices is one of the most striking cortico-cortical connectivities in the primate frontal cortex [52]. Sufficient fMRI and positron emission tomography (PET) studies demonstrated that connectivity exists in these areas in function as well [53, 54]. The connectivity provides powerful avenues of communication between cognitive and motor systems [52, 55]. Two groups that accepted acupuncture stimulation showed the same changes, so we speculated that acupuncture might regulate ACC-relevant cognition and motor activity. The thalamus, insula, ACC, and OFC mainly make up the homeostatic afferent processing network, which represents all aspects of the physiological condition of all tissues in the body and provides crucial sensory input that is essential for maintaining homeostasis [56]. This network encompasses the sensory input entering the thalamus from the brainstem, with projections to the insula, OFC, and ACC [57]. Visceral sensation in humans is a subjective, conscious experience that results from the modulation of homeostatic feelings by cognitive, emotional, motivational factors and memories. Thus, altered visceral perception may be induced by activity changes in the visceral afferent signal processing area. Different studies have confirmed consistent activation of the homeostatic afferent processing network in visceral stimulation. Studies also showed that functional gastrointestinal disorder patients shared alterations in the perceptual and reflex response to homeostatic afferent signals from the gastrointestinal tract [13]. Consequently, we speculated that acupuncture brings functional connectivity changes to the homeostatic afferent network only in FD patients because of their network's functional abnormality.

As mentioned above, ReHo map showed some brain regions involved in the homeostatic afferent processing network and DMN in the FD group changes but not in the HC group after acupuncture. Among these brain regions, change in the ACC is related to clinical symptom alleviation. Moreover, the ACC-related functional connectivity network indicated that the homeostatic afferent processing network only changed in the FD group. This meant acupuncture could regulate the unbalanced organism state, and the homeostasis afferent processing network might play a key role in the central mechanism of treating FD by stimulation at ST25.

Some limitations should be taken into account when interpreting the results of this study. First, the sample size was small (18 subjects for each group). Better results would have been gained with a larger sample. Second, the participants received acupuncture for ten sessions during two weeks. Intestinal symptoms of the FD group were alleviated after treatment, but quality of life did not improve significantly due to such short treatment time. Longer treatment cycle is recommended in future studies.

This study revealed the similarities and differences in resting-state ReHo and the functional connectivity response between the FD patients and HC with the same acupuncture stimulation. Modulation of the sensory, cognition, and motor pathways might be the common mechanism of acupuncture treatment in different groups. Some brain regions only changed in the FD group. More importantly, these changes were negatively correlated with the change in the defecation frequency. Our results demonstrated that acupuncture influenced the left ACC and the homeostatic afferent processing network during the resting state. Our findings hope to shed light on the underlying mechanisms of acupuncture on ST25 for FD.

## Figures and Tables

**Table 1:** Baseline characteristics.

Characteristic	Healthy controls (*n* = 18)	Functional diarrhea (*n* = 18)	*P* value
Age (y)	22.72 ± 1.21	22.24 ± 2.30	0.144
Male/female	10/8	10/8	
Defecation times	6.92 ± 0.81	16.17 ± 5.28	0.000
BSFS score	4.03 ± 0.21	5.58 ± 0.63	0.000
PCS score	57.90 ± 1.45	51.41 ± 6.84	0.000
MCS score	54.47 ± 4.54	45.7 ± 10.78	0.006

BSFS: Bristol stool form scale; PCS: physical component summary; MCS: mental component summary.

Presented values are mean ± SD.

**Table 2:** Clinical outcome measurements pre- and postacupuncture stimulation.

Characteristic	Healthy controls		*P* value	Functional diarrhea		*P* value
	Pre (*n* = 18)	Post (*n* = 18)		Pre (*n* = 18)	Post (*n* = 18)	
Defecation times	6.92 ± 0.81	7.16 ± 0.99	0.144	16.17 ± 5.28	9.78 ± 3.75	0.000
BSFS score	4.03 ± 0.21	4.00 ± 0.19	0.858	5.58 ± 0.63	4.75 ± 0.52	0.001
PCS score	57.90 ± 1.45	57.91 ± 1.62	0.808	51.41 ± 6.84	53.37 ± 4.94	0.085
MCS score	54.47 ± 4.54	55.07 ± 3.99	0.751	45.7 ± 10.78	45.01 ± 7.75	0.711

BSFS: Bristol stool form scale; PCS: physical component summary; MCS: mental component summary.

Presented values are mean ± SD.

## Abbreviations

ACC: Anterior cingulate cortex
DLPFC: Dorsolateral prefrontal cortex
DMN: Default mode network
FD: Functional diarrhea
fMRI: Functional magnetic resonance imaging
FEW: Family-wise error
FGID: Functional gastrointestinal disorders
FWHM: Full width at half-maximum
HC: Healthy controls
ReHo: Regional homogeneity
ROI: Region of interest
KCC: Kendall's coefficient of concordance
MCS: Mental component summary
OFC: Orbital frontal cortex
PCS: Physical component summary
PET: Positron emission tomography
SAS: Self-rating Anxiety Scale
SBFS: Bristol Stool Form Scale
SF-36: MOS 36-item Short Health Survey
SDS: Self-rating Depression Scale
SPM5: Statistical Parametric Mapping 5
TCM: Traditional Chinese medicine.
